# Training Clinicians to Support Aging in Place in a Medicaid Home and Community-Based Waiver Program

**DOI:** 10.1093/geroni/igaa057.1410

**Published:** 2020-12-16

**Authors:** Sandra Spoelstra, Monica Schueller, Alla Sikorskii, Katelyn Ware

**Affiliations:** 1 Grand Valley State University, Grand Rapids, Michigan, United States; 2 Michigan State University, Grand Rapids, Michigan, United States

## Abstract

A randomized trial in 18 home and community-based waiver sites implemented an aging-in-place model for 12,000 disabled older (mean age 78) adults. The trial was underpinned by the Knowledge-to-Action model and utilized 7 implementation strategies, including a 5.5-hour online social worker (SW) and registered nurse (RN) training. Baseline self-efficacy and attitudes of SWs and RNs were measured using validated scales, and knowledge uptake and satisfaction with a tool designed by the team. Characteristics, knowledge uptake, and satisfaction of SWs versus RNs were compared using t-, Wilcoxon, and chi-square tests. Two hundred forty-one RNs and 264 SWs participated. RNs were older (mean age 50; standard deviation [SD] 10.95) than SWs (41.35; SD=11.21) p<.01; and >90% overall were female. RNs had more professional experience, while SWs worked more years in the waiver (p<.01). SWs had greater self-efficacy (t(497)=2.99, p<.04), better attitudes (t(500)=2.59, p<.01), employability (t(500)=2.99, p<.04), and balance (t(491)=2.03, p<.05) than RNs. No differences were found on leadership, organizational culture, motivation, pressure to change, or attitude toward evidence-based practice. Knowledge uptake (range 1-16) was high and did not differ for RNs (Mean=15.2, SD=1.23) versus SWs (Mean=15.26, SD=0.89). Training content, format, role explanation, and information satisfaction (range 0-50) means also did not differ for RNs (Mean=35, SD=10.2) or SWs (Mean=34.9, SD=9.8). While many of the characteristics and outcomes were similar for RNs and SWs, SW’s higher self-efficacy, better attitude, and employability despite less experience in the waiver indicate they may play a positive role in implementation of the intervention that is currently underway.

